# Psychophysiological Associations between Chronic Tinnitus and Sleep: A Cross Validation of Tinnitus and Insomnia Questionnaires

**DOI:** 10.1155/2015/461090

**Published:** 2015-10-25

**Authors:** Martin Schecklmann, Maximilian Pregler, Peter M. Kreuzer, Timm B. Poeppl, Astrid Lehner, Tatjana Crönlein, Thomas C. Wetter, Elmar Frank, Michael Landgrebe, Berthold Langguth

**Affiliations:** ^1^Department of Psychiatry and Psychotherapy, University of Regensburg, 93055 Regensburg, Germany; ^2^Department of Psychiatry, Psychosomatics and Psychotherapy, kbo-Lech-Mangfall-Klinik Agatharied, 83734 Hausham, Germany

## Abstract

*Background*. The aim of the present study was to assess the prevalence of insomnia in chronic tinnitus and the association of tinnitus distress and sleep disturbance.* Methods*. We retrospectively analysed data of 182 patients with chronic tinnitus who completed the Tinnitus Questionnaire (TQ) and the Regensburg Insomnia Scale (RIS). Descriptive comparisons with the validation sample of the RIS including exclusively patients with primary/psychophysiological insomnia, correlation analyses of the RIS with TQ scales, and principal component analyses (PCA) in the tinnitus sample were performed. TQ total score was corrected for the TQ sleep items.* Results*. Prevalence of insomnia was high in tinnitus patients (76%) and tinnitus distress correlated with sleep disturbance (*r* = 0.558). TQ sleep subscore correlated with the RIS sum score (*r* = 0.690). PCA with all TQ and RIS items showed one sleep factor consisting of all RIS and the TQ sleep items. PCA with only TQ sleep and RIS items showed sleep- and tinnitus-specific factors. The sleep factors (only RIS items) were sleep depth and fearful focusing. The TQ sleep items represented tinnitus-related sleep problems.* Discussion*. Chronic tinnitus and primary insomnia are highly related and might share similar psychological and neurophysiological mechanisms leading to impaired sleep quality.

## 1. Introduction

Several neural models regarding the generation and maintenance of chronic and bothersome tinnitus postulate that cochlear dysfunction may be associated with adaptive processes involving both auditory pathway and nonauditory areas (for overview [1]). Patients report difficulties distracting themselves from their tinnitus or negatively appraise their tinnitus, which is in line with the notion of attention (frontoparietal areas) and distress (limbic areas) network involvement in chronic tinnitus [2]. Moreover, increased connectivity between auditory and emotional/autonomic areas has been described in tinnitus patients [3]. An early neurophysiological model suggests that negative emotional and cognitive reaction to the tinnitus percept leads to a distress response of the autonomic nervous system [4, 5]. By mechanisms of conditioned reflexes the tinnitus percept is reinforced by the negative autonomic reaction. These interactions between auditory and nonauditory brain regions can explain why tinnitus is perceived as bothersome and might be the reason why habituation to the tinnitus percept is prevented. These mechanisms can lead to comorbid conditions such as concentration problems, depressivity, and sleep disturbances [6]. Accordingly, insomnia shows an increased prevalence in chronic tinnitus [7, 8] and an impaired sleep quality is correlated with tinnitus distress and sleep quality [9]. Patients frequently report that tinnitus prevents them from falling asleep. Moreover, tinnitus perception and annoyance depends on the quality of the night's sleep and also of afternoon naps. Patients with chronic tinnitus show altered sleep architecture with a higher amount of light sleep as compared to healthy controls [10]. Another study comparing patients with chronic tinnitus and healthy controls showed no differences in polysomnographic sleep parameters except lower nonrapid eye movement spectral power in delta band which was correlated with subjective sleep complaints [11] and lower subjective sleep quality in tinnitus. Insomnia patients with and without tinnitus revealed similar impaired sleep architecture [12]. First trials with sleep-regulating medication such as melatonin showed divergent results [13, 14].

Recently, dysfunctional cognitions, hyperarousal, and increased sympathetic activation were discussed as common etiological factors in comorbid tinnitus and sleep disorders [15]. Primary or psychophysiological insomnia—insomnia without comorbid somatic or psychiatric disorders—is characterised by a physiological hyperarousal, impaired sleep quality, associated tiredness, dysfunctional thinking about sleep, and unfavourable sleeping habits [16]. Recently, parts of the cingulate cortex and bilateral insula were identified as neural substrate for abnormal autonomous activity as elicited by heart rate variability, which in turn is mediating tinnitus distress [17].

Based on the idea of common etiological factors in chronic tinnitus and psychophysiological insomnia [15], the aim of the present study was to assess the prevalence of insomnia in chronic tinnitus and the association of tinnitus distress and sleep disturbance. Furthermore, we evaluated whether the Tinnitus Questionnaire (TQ [18]) is sufficient for screening of insomnia symptoms in chronic tinnitus. For this purpose a retrospective analysis of the association of the TQ and the Regensburg Insomnia Scale (RIS [12]) of a large sample of patients with chronic tinnitus was performed. The TQ is a validated questionnaire for the assessment of tinnitus severity with a well-established factorial structure. Among the 6 components one factor assesses specifically sleep problems. The RIS was specifically established for the assessment of psychological factors related to primary/psychophysiological insomnia [19].

## 2. Materials and Methods

All included subjects were patients of the in- or outpatient clinic of the Department of Psychiatry and Psychotherapy. Tinnitus was diagnosed in the outpatient clinic which is part of the Interdisciplinary Tinnitus Center at the University of Regensburg (Regensburg, Germany). Patients gave written informed consent for data collection in the Tinnitus Research Initiative database [20] which was approved by the Ethics Committee of the University Hospital of Regensburg (Germany; reference number 08/046). Inclusion criterion was subjective chronic tinnitus. Exclusion criteria were objective tinnitus (with a treatable cause) and presence of unstable psychiatric comorbidities or unstable medical conditions. Patients were 53 ± 11.1 years old; 129 out of 182 (71%) were men, and had a tinnitus duration of 98.2 ± 99.2 (*n* = 171) months and tinnitus distress level of 46.3 ± 18.0 as indicated by the TQ. Seventeen out of 177 showed purely right, 32 purely left tinnitus and 128 tinnitus in both ears or within the head.

Patients filled in the German versions of the Tinnitus Questionnaire (TQ [18, 21]) and the Regensburg Insomnia Scale (RIS [19]). The TQ total score is the summation of 40 items with 2 items counted double. The different subscales are emotional distress, cognitive distress, sleep disturbance, auditory perceptual difficulties, somatic complaints, and intrusiveness. To control for effects of sleep items in the TQ total score we subtracted the sleep subscore from TQ total score for the statistical analyses.

For statistical analysis we present descriptive comparisons of the present tinnitus sample with the insomnia sample of the validation study of the RIS [19]. The validation sample was recruited and carefully diagnosed in the Center for Sleep Medicine at the Department of Psychiatry and Psychotherapy of the University of Regensburg (Germany). We correlated the RIS sum score with the TQ scales using Pearson correlation coefficient. Contrasts between correlations were done by using Fisher's *r*-to-*z* transformation. Two principal component analyses (PCAs) with Varimax rotation were used to test for the homogeneity of the TQ sleep and the RIS items. For the first PCA all TQ items and all RIS items were included; for the second PCA only TQ sleep items and the RIS items were included. To test for the homogeneity between tinnitus and insomnia patients we recalculated the PCA of the RIS validation study for the present tinnitus sample by using only the RIS items. For factor extraction we used the Kaiser-Guttman criterion (eigenvalues > 1). According to Hair et al. [22] items with factor loadings above 0.45 were included for the factor solution. Statistical analyses were performed with SPSS 18.0.0 (SPSS, USA).

## 3. Results

138 out of 182 tinnitus patients (76%) presented with insomnia as categorised by the cut-off score of the RIS (>12). The mean RIS score (17.5 ± 7.6) was about five points below the mean RIS score of the validation sample [22] which included only patients with primary insomnia. On single item level the difference between the two samples was in the same range except for the items nine and ten. With respect to those two items (“*daytime functioning*” and “*medication intake*”) tinnitus and insomnia patients had similar values (Table 1).

Tinnitus patients showed significant medium to high correlations of the RIS sum score with the total score of the TQ (corrected for the sleep items) and all subscores (Table 2). The correlation of the RIS with the sleep subscore was the highest one. The correlation between the RIS and the TQ sleep subscore was significantly higher than the correlation of the RIS with the other TQ scores (Table 2).

Principal component analyses with all TQ and RIS items fulfilled the statistical requirements (Kaiser-Meyer-Olkin measure: KMO = 0.905; Bartlett's test: *p* < 0.001) and showed ten factors including one sleep factor consisting of three of the TQ sleep items and the RIS items 2–8 (Table 3). Principal component analyses with all sleep items (four TQ sleep items and all RIS items) fulfilled the statistical requirements (Kaiser-Meyer-Olkin measure: KMO = 0.900; Bartlett's test: *p* < 0.001) and showed three factors. Factor one consisted of the RIS items 2–6 (sleep depth), factor two consisted of all TQ sleep items (tinnitus-related sleep), and factor three consisted of RIS items 1, 7, and 8 (fearful focusing and sleep onset). Principal component analyses with only the RIS items fulfilled the statistical requirements (Kaiser-Meyer-Olkin measure: KMO = 0.871; Bartlett's test: *p* < 0.001) and showed two factors. Factor one consisted of the RIS items 2–6 (*sleep depth*) and factor two consisted of items 7 and 8 (*fearful focusing*). The factors* fearful focusing* and* sleep depth* were also extracted in the insomnia validation sample whereas the other extracted factors from the validation sample (*sleep quantity* and* sleep medication/daytime functioning*) could not be extracted in our sample.

## 4. Discussion

Our retrospective correlation analysis of Tinnitus Questionnaire (TQ) and Regensburg Insomnia Scale (RIS) in 182 patients with chronic tinnitus showed three main results.

(1) According to the cut-off of the RIS sum score 76% of all tinnitus patients in our sample suffer from insomnia. This finding emphasizes that insomnia represents a major problem in chronic tinnitus. With respect to daytime functioning and sleep medication intake tinnitus patients display similar scores as patients with primary insomnia. The correlation analyses revealed a rather high association (*r* = 0.558) between tinnitus distress (TQ sum score corrected for the sleep items) and sleep disturbance (RIS score). This finding is in line with recent results of a significant correlation of 0.62 in 117 US tinnitus patients between the Tinnitus Reaction Questionnaire and the Insomnia Severity Index [23]. In another study of 97 veterans with tinnitus a small association of 0.214 between the Tinnitus Handicap Inventory (measuring tinnitus distress) and the Epworth Sleepiness Scale (measuring daytime sleepiness) was found [9]. Taken together these findings confirm consistently that sleep problems have an impact on tinnitus severity, but they also show that the exact magnitude of the relationship depends on sample selection and assessment instruments.
(2) The TQ sleep subscore is highly correlated with the RIS. Tinnitus-related sleep disturbance as indicated by the TQ explains about 48% (*r* = 0.690) of the variance of psychophysiological sleeping problems as indicated by the RIS which was constructed for measuring psychological aspects of insomnia. The PCA with all items of the TQ and RIS resulted in a common factor “*sleep*” including all RIS items and all four sleep items of the TQ. Thus, the TQ with its subscore structure seems to be sufficient as a screening tool for tinnitus-related insomnia. However, more detailed insights into the association between sleep and tinnitus are necessary for a more effective clinical management of tinnitus patients with insomnia. It remains unclear if the vicious circles of insomnia and tinnitus are connected by common symptoms such as dysfunctional beliefs or hyperarousal. It is also an open question if daytime tinnitus annoyance is dependent on sleep quality, sleep quantity, afternoon nap, or kind of sleep stage during awakening. Whereas clinical experience suggests such relationships, specific studies addressing these questions are still lacking. There is evidence that sleep deprivation, selective sleep interruption, and awakening in different sleep stages exert influences on experimentally induced pain [24, 25]. In light of the overlap in neurophysiological mechanisms of chronic pain and tinnitus [26], similar mechanisms could also hold for tinnitus.
(3) Based on the PCAs using only sleep items it turned out that fearful focusing and lack of sleep depth are the two main sleep-related problems in chronic tinnitus. Thus, tinnitus patients and those suffering from psychophysiological insomnia share similar sleep-related problems. In our clinical experience dysfunctional beliefs, negative thoughts, and hyperarousal are encountered in patients with both primary insomnia and chronic tinnitus. However, in tinnitus patients the focus of negative thoughts and emotions is related to both tinnitus and sleep and not to sleep alone. This may be the reason for subjectively experienced longer sleep onset latencies in tinnitus patients with insomnia in contrast to insomnia patients alone [12]. From a clinical perspective, it is to be tested if cognitive behavioural therapy for primary insomnia [27] could also be effective in chronic tinnitus with insomnia [28].

The big limitation of this study might be based on sample bias. As our sample derives from a tertiary referral center and our tinnitus center is known supraregionally, the sample is probably not representative and is rather limited to high-burdened patients although the mean tinnitus distress level was medium.

## 5. Conclusions

From a methodological point of view, our results suggest that the TQ sleep subscore is a good approximation of general sleep-related problems in tinnitus. However, more detailed information can be obtained by using specific questionnaires such as the RIS. Taking into account the specific single items of both examined questionnaires, psychological and physiological mechanisms seem to be similar in chronic tinnitus and primary insomnia. We suggest that future studies should focus on the underlying neurobiological mechanisms of disturbed sleep in tinnitus and also of sleep-mediated tinnitus annoyance.

## Figures and Tables

**Table 1:** Characterization of the sleep items of the Regensburg Insomnia Scale and the Tinnitus Questionnaire sleep subscore items and comparison of the mean scores of the present tinnitus sample with the validation sample of the Regensburg Insomnia Scale.

Items		Insomnia sample	Tinnitus sample	Difference score
		*n* = 218	*n* = 182	
	*Regensburg Insomnia Scale *			
1	Sleep onset latency	1.82 ± 1.37	1.21 ± 1.13	0.61
2	Sleep duration	1.65 ± 0.86	1.02 ± 0.88	0.63
3	Disturbed sleep	3.11 ± 0.97	2.27 ± 1.2	0.84
4	Early awakening	2.86 ± 1.03	2.25 ± 1.09	0.61
5	Awaking by sounds	2.77 ± 1.08	2.25 ± 1.07	0.52
6	Feeling of no night sleep	2.07 ± 0.97	1.53 ± 1.04	0.54
7	Thinking about sleep	2.41 ± 0.92	1.79 ± 1.08	0.62
8	Afraid of going to bed	1.89 ± 1.24	1.48 ± 1.25	0.41
9	Daytime functioning	2.31 ± 1.03	2.12 ± 1.09	0.19
10	Medication intake	1.7 ± 1.56	1.52 ± 1.67	0.18
	*Tinnitus Questionnaire *			
4	Awakening more often due to tinnitus			
12	Early awakening due to tinnitus			
31	Sleep as general problem			
36	Sleep onset latency due to tinnitus			

**Table 2:** Correlation analyses between RIS (Regensburg Insomnia Scale) sum score and TQ (Tinnitus Questionnaire) scores. Please note that TQ total score is corrected for the sleep subscore.

	RIS sum score	Correlation differences of TQ-RIS with RIS-TQ sleep score
TQ score sleep disturbance	*r* = 0.690; *p* < 0.001	—

TQ total score	*r* = 0.558; *p* < 0.001	*z* = 2.06; *p* = 0.039

TQ score cognitive distress	*r* = 0.545; *p* < 0.001	*z* = 2.24; *p* = 0.025

TQ score emotional distress	*r* = 0.452; *p* < 0.001	*z* = 3.41; *p* < 0.001

TQ score intrusiveness	*r* = 0.450; *p* < 0.001	*z* = 3.44; *p* < 0.001

TQ score auditory perceptual difficulties	*r* = 0.371; *p* < 0.001	*z* = 4.34; *p* < 0.001

TQ score somatic complaints	*r* = 0.462; *p* < 0.001	*z* = 3.21; *p* = 0.001

**Table 3:** Factor solutions for the sleep components using principal component analyses (PCAs).

	PCA with all TQ and RIS items	PCA with only TQ sleep and RIS items			PCA with only RIS items	
	Factor 1	Factor 1	Factor 2	Factor 3	Factor 1	Factor 2
	Sleep	Sleep depth	Tinnitus-related sleep	Fearful focusing and sleep onset	Sleep depth	Fearful focusing
*RIS items *						
1	(0.357)			0.570		(0.434)
2	0.693	0.491			0.667	
3	0.705	0.686			0.799	
4	0.688	0.604			0.610	
5	0.628	0.556			0.553	
6	0.696	0.666			0.595	
7	0.606			0.507		0.638
8	0.542			0.531		0.774
9						
10						
*TQ items *						
4	0.531		0.768			
12	0.538		0.771			
31	0.582		0.537			
36	(0.389)		0.594			

Values in brackets lie below the a priori defined factor loading threshold of 0.45.
